# Models of reablement evaluation (MoRE): a study protocol of a quasi-experimental mixed methods evaluation of reablement services in England

**DOI:** 10.1186/s12913-016-1600-6

**Published:** 2016-08-11

**Authors:** Rachel Mann, Bryony Beresford, Gillian Parker, Parvaneh Rabiee, Helen Weatherly, Rita Faria, Mona Kanaan, Alison Laver-Fawcett, Gerald Pilkington, Fiona Aspinal

**Affiliations:** 1 Social Policy Research Unit, University of York, York, YO10 5DD UK; 2 Centre for Health Economics, University of York, York, YO10 5DD UK; 3 Department of Health Sciences, University of York, York, YO10 5DD UK; 4 Faculty of Health and Life Sciences, York St John University, York, YO31 7EX UK; 5 Gerald Pilkington Associates, 74 Poplar Grove, New Malden, KT3 3DN UK

**Keywords:** Reablement, Restorative, Home care, Health care, Social care, Mixed methods evaluation, Study protocol

## Abstract

**Background:**

Reablement is a time-limited intervention that aims to support people to regain independence and enable them to resume their daily activities after they return home from an in-patient care setting, or to maintain independence to enable them to remain at home. There is some evidence that reablement can enhance independence and has the potential to contain costs. However, reablement services are funded and provided in different ways and by different organisations, and there is limited research evidence about the effectiveness of different reablement service models. This study will evaluate the effectiveness and cost-effectiveness of different reablement service models and service users’ and carers’ experiences of reablement in England, UK.

**Methods/Design:**

The study will use a quasi-experimental mixed methods design that comprises three work packages (WP) extending over a period of 34 months. WP1 will conduct cluster analysis on survey data to develop a typology of current models of reablement services in order to describe the current reablement service landscape. WP2 will comprise a quantitative outcomes evaluation of the effectiveness of the different service models; a process evaluation and an economic evaluation. WP2 will be set within generic reablement services, where providers are using the most commonly employed generic reablement service types identified in WP1; the primary outcome measure is health-related quality of life measured by the EQ-5D-5L. WP3 will provide evidence about specialist reablement services and how specialist approaches and practices are organised and delivered.

**Discussion:**

Managing demands on care services is, and will remain, a crucial factor for the UK National Health Service as the number of people with long-term conditions rise. There has been, and will continue to be, significant investment in reablement services. The proposed study will address several key areas where there is limited evidence regarding the organisation and delivery of reablement services in England, UK. Specifically, it will provide new evidence on different models of reablement services that will be of direct benefit to health and social care managers, commissioners and their partner organisations.

**Electronic supplementary material:**

The online version of this article (doi:10.1186/s12913-016-1600-6) contains supplementary material, which is available to authorized users.

## Background

A rapidly ageing population and advances in technology have led to a dramatic rise in the number of people living longer with long-term conditions that affect their ability to live independently [1, 2]. This has resulted in a significant rise in hospital admissions and increasing pressure on social care services including intermediate care and community services [3]. Improvements in care in some childhood conditions have led to increasing numbers of young people with complex needs surviving into adulthood [4]. Attempts to bring about a real shift to community-based care and avoid unnecessary/lengthy hospital admissions or ‘unnecessary dependency’ on social care services have long dominated health and social care policies and strategies in the UK and other countries [5–7].

Reablement is a relatively new approach that aims to support people to regain or maintain independence in their daily lives [8]. The reason for referring an individual to a reablement service can be conceived as falling into one of two broad categories: to support an individual to return home from hospital or other in-patient care setting following an acute episode; or to support an individual to remain at home, with minimum demands on home-care/community services, where there is evidence of declining independence or ability to cope with everyday living [9]. Reablement is a time-limited intervention to help enable people to resume the everyday activities which make up their daily lives for example, cleaning the house, shopping or bathing and dressing themselves independently rather than having someone (such as an informal or formal carer) do things ‘to’ them or ‘for’ them [8, 9].

The key characteristics that have been identified [10], which distinguish reablement from other health and social care services are, the time-limited nature of the service, that it contains a restorative/self-care element and that it aims to achieve some (or all) of the following five service objectives: acute admission avoidance at the point of clinical need for acute care; early supported discharge after acute admission; longer-term avoidance of unplanned hospital admission; reduction in the use of home care services; avoidance of admission to long-term care.

The UK government has invested substantially in reablement services as there is some evidence that they can enhance independence and have the potential to contain costs [11]. This was signalled by £70 million extra funding made available to the UK National Health Service (NHS) in 2010 to support hospital discharge [12], followed by £300 million/year over 2012–15 for ‘reablement spending’ [13]. More recently, the UK government announced an investment of £91.6 billion in local NHS services in 2012/3, including £150 million for reablement [14]. The promotion of reablement services within the Care and Support 2012 White Paper has also firmly established it as a priority for local authorities [15].

While existing research suggests that reablement can lead to improvements in health-related quality of life, social care outcomes and physical functioning and remove or decrease the need for commissioned health and home care services [11, 16], the evidence base around reablement services remains limited and significant questions remain. The rapid development of reablement services in England (UK) has created variation in the service landscape. Differences in how services are funded and delivered by different organisations and their effectiveness on service user outcomes cannot be addressed by the existing body of research in this area [11]. Reablement is reported to work differently for different people, being less effective for those with chronic/complex health problems compared to people recovering from acute illnesses or falls [17]. Considerable variation is also reported in local arrangements for self-care services for long-term conditions [18]. Indeed, for some groups (e.g. young adults with life-limiting conditions and adults with dementia), the evidence base is virtually non-existent. Evidence suggests that the number and proportion of people with specialist needs is growing fast. For example, the number of people living with dementia in the UK is expected to double in the next 30 years while the overall costs of dementia in the UK are estimated to triple from £17 billion per year to over £50 billion [19]. The rapid rise in the number and proportion of people with specialist needs suggests that responding to such needs will become an ever-growing pressure on health and social care services. It is, therefore, important that both those populations that access ‘generic’ reablement services as well as those with specialist needs are represented in this research.

This study will evaluate: whether the type of service model affects services users’ outcomes; what other factors, such as age and other medical conditions, affect the effectiveness and cost-effectiveness of different reablement service models; and service users’ and carers’ experiences of reablement.

## Methods/Design

The primary aim of this study is to evaluate different models of reablement services, which will generate evidence to support commissioners and practitioners as they make decisions about the organisation, delivery and development of reablement services. The study comprises three work packages using different methods and data collection strategies extending over a period of 34 months. These are:

- Work package 1 (WP1): Mapping reablement services.
- Work package 2 (WP2): Evaluation of generic reablement service models.
- Work package 3 (WP3): Description of specialist reablement services/practice approaches.

To avoid confusion between the use of the term ‘model’ to describe service configuration (i.e. service model) and statistical techniques (i.e. modelling), the term ‘service type’ has been used instead of ‘service model’ throughout the methods section.

### Work package 1 (WP1): mapping reablement services

Work package 1 (WP1) will generate ‘stand-alone’ evidence on reablement services in England and develop a typology of current types of reablement services. The typology will be used to identify four service types for evaluation. It will also be used to fine-tune the design and sampling of WP2 and WP3. It will employ survey methodology comprising a three-stage process: a) identification of reablement services; b) identification of key informants in each reablement service; and c) data collection from key informants of reablement services.

### Methods – WP1

- Identification of reablement services
  The first stage of WP1 will involve an initial screening exercise to identify the person most closely involved with commissioning intermediate care/reablement services in every Clinical Commissioning Group (CCG) (*n =* 221) or Local Authority (LA) with responsibility for adult social services (*n =* 154) in England. Some of these key informants may commission joint services, others may have more than one service, and a few may have none, so the total number of *services* surveyed is expected to be approximately 375. The people identified as the key informant in each service will be contacted by telephone and asked about services that they commission that serve any, some or all of the five objectives of intermediate care/reablement [10].
- Identification of key informants in each reablement service
  Individuals identified as the key informant most closely involved with commissioning intermediate care/reablement services in every CCG or LA with responsibility for adult social services that serve any, some or all of the five objectives of intermediate care/reablement [10] will be asked to provide contact details for the managers of these services. Managers of the service will be contacted by telephone to: confirm the objectives of their service; that they operate in a time-limited form; and include some element of restorative input. This will establish whether the manager identified by the commissioner is the person best placed to answer detailed questions about the service delivery and organisational characteristics of the service.
- Data collection from key informants of reablement services
  A structured questionnaire will be administered using electronic survey software to each of the identified key informants of each reablement service. It will cover a range of service delivery and organisational characteristics that research with service managers, practitioners and users suggests are important in influencing outcomes in these types of services [11, 20, 21]]. Where there are cases of non-response or where answers are unclear or ambiguous, respondents will be followed-up via telephone contact.

#### Analysis

Survey data will be analysed descriptively as a first stage, to give a simple national picture of current reablement provision. Cluster analysis [22] will be used to develop a typology of reablement services using data on the characteristics of the services. The service delivery and organisational variables collected from the survey will form the basis for analysis. Cluster analysis is a useful way of developing a tight typology of services in order to assist analysis of differences and similarities between services, while at the same time preserving the underlying features of individual services. The typology of services will be tested using bivariate analysis to ensure that it does differentiate between different types of reablement service types. A second cluster analysis will also be undertaken using the survey data on case mix, size and eligibility, to categorise the different service user groups served by reablement services. The results of the cluster analysis will stand in their own right as a typology of reablement services but will also inform sampling of services for the next stage of the research, when the study will focus on the differential impact of specific service types.

WP1 has been completed and four generic reablement service types have been identified for evaluation in Work package 2. The findings from WP1 will be reported elsewhere.

### Work package 2 (WP2): evaluation of generic reablement services

Work package 2 (WP2) comprises three elements: an outcomes evaluation (WP2a); a process evaluation (WP2b); and an economic evaluation (WP2c).

### Outcomes evaluation (WP2a)

WP2a outcomes evaluation is a quantitative evaluation of the effectiveness of the different service types, which will examine short and longer-term service-level and individual-level outcomes, and compare outcomes between service types. Quantitative patient-reported and practitioner-reported measures will be used to understand the effects of reablement and the impact of service and user characteristics and other factors on effectiveness.

### Methods – WP2a

#### Setting

WP2a will evaluate four types of reablement services identified in WP1 above. WP2a will be set within generic reablement services, where providers are using the most commonly employed generic reablement service types across England (UK) as identified in WP1. This should ensure a meaningful and applicable evaluation with findings transferable to other settings.

#### Participants – inclusion/exclusion criteria

People referred to reablement services aged 18 and over (no upper age limit is imposed) are eligible to take part in the study if they have been offered and accepted a programme of reablement, and are able to give written informed consent. There are no study exclusion criteria other than lack of capacity to give informed consent [23].

#### Data collection

Service users recruited to the project will be the main source of data collection. Individual participant outcomes will be measured at three time points: within the first week of receiving the reablement service (T_0_); within a week of discharge from the reablement service (T_1_); and 6 months post-discharge (T_2_ = T_1_ + 6 months).

In addition, practitioners conducting reablement service assessments and reablement practitioners will be asked to collect background data on health, functioning and living situation. For each research participant, the reablement practitioner conducting the reablement service assessment (T_0_) will complete a study entry form and information about functional status, which includes questions on: health status; impairments (e.g. memory/confusion; cognitive impairment); living situation (e.g. living alone versus with partner/other family) and informal carer involvement. Services will also be asked to provide the study with routinely collected service delivery/audit data.

#### Outcome measures

Service-level and individual-level outcomes will be examined to explore how these vary within and between reablement service type and by individual characteristics.

#### Individual participant level outcomes

Outcome measures have been selected which together capture health, well-being, social inclusion, participation and self-efficacy. In addition, attainment of user-specific reablement goals will be recorded.

#### Primary outcome measure

The EQ-5D-5L questionnaire will be the primary outcome measure used to measure health-related quality of life [24, 25]. It represents five dimensions of quality of life: mobility, self-care, usual activities, pain/discomfort and anxiety/depression. Respondents indicate the level of difficulty they are experiencing in each of these domains on a five point scale (no problems, some problems, moderate problems, severe problems, unable).

#### Secondary outcome measures

The Adult Social Care Outcomes Toolkit (ASCOT SCT-4) [26, 27] will be used to measure social-care related quality of life. It measures social care quality of life across nine domains: control over daily life; personal cleanliness and comfort; food and drink; personal safety; social participation and involvement; occupation; accommodation cleanliness and comfort; dignity. The ASCOT tools are used routinely by local authorities and government and were used in a previous national evaluation of reablement services in England [11].

#### General health questionnaire (GHQ-12)

The GHQ-12 is a measure of current mental health [28]. It focuses on two major areas—the inability to carry out normal functions and the appearance of new and distressing experiences. It is well suited for longer-term studies that require an indicator of minor psychiatric morbidity [29].

#### User-defined goal attainment

It is routine practice for quantifiable reablement goals to be agreed between practitioners and individuals when the reablement assessment process takes place [11]. We will record the goals set with each participant and determine goal attainment as per the service method when the participant is discharged from the service.

#### Service level outcomes

The reason for referring an individual to a reablement service can be conceived as falling into one of two broad categories: to support an individual to return home (RH) from hospital or residential care setting following an acute episode or to support an individual to remain at home (RAH). Service level outcomes will include the proportion of RH users readmitted to hospital within six months of discharge from the reablement service; the proportion of RAH service users placed in residential care within six months of discharge from the reablement service; and the proportion of RAH service users with reduced use of ‘home care’ type services at six months after discharge from the reablement service.

#### Other measures

##### Experiences of reablement practice

At discharge (T_1_), research participants will complete a brief questionnaire about their views on the practice and approach of the reablement practitioners who worked with them. This will cover their perceptions of their relationship with reablement practitioners and their views on the way practitioners worked with them. There are no pre-existing questionnaires or measures which captures this concept, therefore the research team will develop and pilot a questionnaire in Year 1 of the study.

##### Engagement in reablement

At discharge (T_1_), reablement practitioners will complete a measure of user engagement in reablement. There are no pre-existing measures of engagement in reablement and therefore the Hopkins Rehabilitation Engagement Rating Scale (HRERS) will be adapted for this study with permission from the authors [30]. The HRERS was developed to measure service user engagement in acute rehabilitation services. Some adaptations are required to make it suitable for use in a reablement context. Specifically, the word ‘therapy’ will need to be replaced with a suitable alternative and the first item concerns attendance rates at outpatient clinics, so these will need to be adapted to reflect delivery of reablement services in the client’s home. During Year 1 of the study a consultation will be undertaken with rehabilitation practitioners regarding adaptations.

#### Sample size

The statistical approaches chosen to measure outcomes takes into account the potentially clustered nature of the data collected.

The marginal effect of reablement versus home care as usual in a previous prospective longitudinal study was 0.107 [11]. This was taken as comparable to Cohen’s f^2^ [31] and indicative of a small to medium effect size. It was assumed that differences in effect size between different types are likely to be smaller than those seen between reablement versus usual care and have, therefore, used the relatively small effect size of f^2^ = 0.06. Using a sample size calculation for hierarchical multiple regression, without clustering, the minimum total sample size required to detect an effect size of 0.06 with a power of 80 % and an alpha of 0.05, and with up to twenty individual predictors and four reablement service type predictors would be 222.

In order to account for clustering in the sample size calculations, two reported estimates of intra-class correlations (ICC) and the associated design effects have been used [32]: i) a reported ICC of 0.007 for overall health with an average cluster sample size of 200; this would result in a design effect of 2.393; and ii) the highest reported ICC of 0.01355 in the study [32]. This was deemed necessary for the sample size estimate as people eligible for reablement are likely, as a group, to display less variation in overall health (and therefore a higher ICC). The ICC of 0.01355 was rounded to 0.014 and, using an average cluster size as above, results in a design effect of 3.786.

Inflated by the two design effects given above (2.393 and 3.786), the study will need to achieve a sample size of between 532 and 841. Assuming an attrition rate of 25 % between T_0_ and T_2_, the study will need to recruit between 710 and 1121 people in total. A pragmatic decision was made to recruit between these two figures, giving a sample of 800 people in total, around 200 per each of the four reablement service types to be evaluated in WP2.

#### Analysis

Baseline (T_0_) demographic and social characteristics of participants at each case site will be summarised using descriptive statistics. Categorical data will be reported as counts and percentages. Continuous data will be expressed as mean values with standard deviations, however median and inter quartile range will be reported if the data is skewed. Multilevel modelling has been chosen as the most appropriate analytical approach for this type of study design, which allows exploration of more than one explanatory variable at different levels and facilitates analysis of cross-level interactions [33]. This will allow separate analysis of the impact of service user characteristics, the types of reablement service, and time on service user outcomes, and identification of any possible interactions between them. This approach is thus ideally suited to exploring questions of effectiveness and which sub-groups of service users might benefit most.

### Process evaluation (WP2b)

A process evaluation, comprising interviews with reablement professionals, service users and carers, will develop an understanding of the immediate and wider context in which reablement models exist, the different effects reablement can have and how, and why, these effects vary between recipients and different services.

### Methods - WP2b

A mixed-methods [34] approach will be used for the process evaluation. Data collected in WP1 will be used alongside data from qualitative interviews with commissioners, practitioners, service users and carers. These semi-structured interviews will allow more in-depth exploration of local policy, structures and process arrangements, the components of the service, service costs, how these components interact and the effects of these interactions.

The interviewing process will take a staged approach. In order to understand local structures, systems and service delivery/practice issues interviews will be conducted with lead commissioners, service managers and practitioners. Interviews will be conducted with service users and carers to understand their experience of reablement and goal achievement (*n =* 10 to 12 per service type). Further interviews with reablement practitioners (*n =* 6 to 8 per service type) will also be conducted to identify the barriers and facilitators to achievement of service user outcomes.

#### Sampling and recruitment

##### Staff

The aim is to recruit the lead commissioner/s for reablement and the reablement service manager/s within each of the four service types as described in WP2a. Practitioners who deliver the reablement programmes to service users will also be interviewed; this includes interviews (with service users’ permission) with the reablement practitioner supporting service users recruited to the process evaluation. The number of practitioners interviewed will depend on the size and structure of the team (this will be unknown until case sites are confirmed following WP1 cluster analysis). However, recruitment from these groups of staff will be undertaken and the views of different professional groups and grades within the team will be gathered until data saturation is achieved.

##### Service users

The aim is to recruit 10–12 service users per service type. Service users will be identified from the outcomes evaluation sample when they are discharged from the reablement service. Half of the service user sample will be ‘return home’ (RH) clients and the other half will be ‘remain at home’ (RAH) clients. Purposive sampling will be undertaken within these two groups to include, for example, different levels of goal achievement and personal and socio-demographic factors that affect goal achievement (based on evidence from staff interviews and preliminary analysis of data collected at discharge, i.e. T_1_ outcomes data).

##### Carers

Interviews will be undertaken with up to ten carers in each case site. Initially carers of service users taking part in the process evaluation will be recruited, but we are aware that some service users might not have a carer. Therefore, carers of other recipients of reablement services will be recruited if needed. Carers will be over 18 years of age and able to give informed consent. Both carers supporting service users in the (RH) group and in the (RAH) group will be recruited.

#### Analysis

Data will be analysed within, and across, participant and reablement service types using the Framework approach to managing and thematically analysing qualitative data. The Framework approach facilitates systematic data management and allows audit trails of the data management process [35].

### Economic evaluation (WP2c)

The economic evaluation will use data collected in WP1 and WP2 to compare the costs and consequences of the four different generic reablement service types in comparable populations to indicate which offers the best value for money. It will comprise: a rapid systematic review [36]; a cost-effectiveness analysis from the perspective of the UK NHS; a cost-consequence analysis taking a societal perspective; the development of a decision analytic model and costing vignettes.

#### Outcomes

The cost-effectiveness analysis will consider health outcomes expressed in quality-adjusted life years (QALYs) using EQ-5D-5L collected during WP2a. The cost-consequence analysis will consider QALYs and social care-related quality of life, using the ASCOT SCT-4 collected during WP2a. Both EQ-5D-5L and ASCOT SCT-4 will be valued using the UK population tariffs [37, 38].

#### Resource use and costs

Resource use will be collected directly from study participants using a bespoke questionnaire, the Service and Care Pathway Questionnaire (SCPQ), collected at T_0_, T_1_ and T_2_. The SCPQ will collect data on the use of health, social care and voluntary services, both publicly funded and privately paid, and informal care. The SCPQ will be piloted with a sample of service users and amended as required prior to the commencement of data collection. Study participants will be asked for their use of hospital services over the previous 6 months. The recall period for other services will be subject to the results of the pilot.

Resource use will be valued with national costs whenever possible. For the services where national costs are not available, the process evaluation in WP2b will obtain the relevant local costs. The cost-effectiveness analysis will include the costs from the perspective of the UK NHS and publicly funded social care. The cost-consequence analysis will include costs falling on all sectors, namely UK NHS, publicly funded social care, voluntary services, private costs and informal care.

#### Decision analytic model

The cost-effectiveness and cost-consequence analyses will involve the development of a decision analytic model to compare the costs and outcomes of the different service types over the longer term and incorporate external evidence on reablement.

#### Costing vignettes

‘Costing vignettes’ will be developed which describe and quantify the care pathway reflecting the sequence of services utilised along the care pathway following reablement The vignettes will report on the context in which the care is provided including any use of informal care, who provides the services and who pays for them. Vignettes will be selected from the cohort of reablement care patients who were tracked for the duration of the study (T_0_-T_2_).

### Work package 3 (WP3): description of specialist reablement services/practice approaches

The aim of this work package is to explore the organisation and delivery of reablement support for people with specialist needs. This includes ‘adapted or extended practice’ within generic reablement services and specialist reablement services. It will build on, and develop, the high level descriptive information collected during WP1. It will also utilise data collected and findings from the process evaluation (e.g. professional interviews in WP2b) in order to explore differences in service types, practice and workforce characteristics between generic and specialist reablement provision.

### Methods - WP3

#### Identification of services—specialist services/practice

One of the outputs of WP1 will be descriptive report of reablement provision across England, including specialist practice within generic teams or specialist/population specific teams. Scrutiny of WP1 data will inform the specifics of the sampling frame. It is therefore, not clear at this stage what type of speciality groups WP3 will focus on or whether the focus will be on more than one type of speciality. The decision about which specialist services to investigate will be informed by the study steering committee, comprising reablement service managers and commissioners, and their guidance about the most pressing concerns related to particular ‘specialist’ populations. However, drawing on the issues and questions set out in the UK National Institute of Health Research (Health Services & Delivery Research) commissioning brief, these might include specialist reablement provision for older people with dementia, young adults with complex needs recently transferred from children’s services, and users with learning disabilities.

#### Sampling and recruitment

It is anticipated that approximately ten services will be investigated. The research sites selected for WP3 will not be participating in WP2. All research sites will be offered the option of anonymous involvement. The aim is to recruit 10 reablement managers working in a specialist team or who work in a generic team with specialist practice. Each manager from these specialist services will also be asked to identify front-line staff with different grades and experiences. From this sample, two front-line reablement practitioners will be recruited per site—producing in total a sample of 20 reablement practitioners across the sites.

#### Data collection

All interviews will be semi-structured and will be conducted over the telephone. These interviews will use similar interview schedules to those used in the process evaluation of generic reablement services in WP2b. This will allow comparative analyses to be undertaken across the two datasets. In addition, the interviews with service managers and front-line staff in WP3 will explore team characteristics, the skill mix for addressing specialist needs and specialist/additional training.

#### Data analysis

As with WP2b, analysis of the data will be thematic, informed by the research questions and use the Framework approach [35].

## Discussion

A quasi-experimental study design was adopted for the Models of Reablement Evaluation (MoRE) study, as given the current service landscape where every local authority in England have reablement services and everyone who is entitled to it is offered reablement, a randomised controlled trial, where participants are randomly assigned to different interventions or a control group that does not receive the intervention, would be unfeasible. This comprehensive evaluation takes advantage of a mixed methods design that contains three important and distinctive work packages that will provide robust quantitative and qualitative evidence regarding the effectiveness and cost-effectiveness of reablement service types.

WP1 will generate evidence on reablement services in England and develop a typology of current models or types of reablement services in order to describe the current reablement service landscape. WP2 will provide comprehensive evidence about the effectiveness and cost-effectiveness of reablement services in the form of a quantitative evaluation of the effectiveness of the different service types. It will also include: a process evaluation to develop an understanding of the immediate and wider context in which reablement models exist; what the different effects reablement can have; how and why these effects vary between recipients and different services/sites; and an economic evaluation to compare the costs and consequences of different generic reablement models in comparable populations. WP3 will provide an initial evidence base for how specialist reablement services and specialist approaches/practice within generic services are organised and delivered to groups with complex or specialist needs. The policy interest in this area is high [19] and there is appetite for information on the features of the organisation and delivery of the intervention that are likely to optimise self-care and independence.

Reablement is highly topical and there has been, and will continue to be, significant investment in reablement services. As the numbers of people with long-term conditions rise and public finance constraints persist, managing demands on care services will remain a crucial factor for the NHS. The rapid move towards development of reablement services that are funded and provided in different ways and by different organisations has created a knowledge gap that cannot be addressed by the existing body of research in this area. This new research is needed to explore issues not addressed in the existing, limited evidence base. Specifically, the MoRE study will explore which models of interventions are more effective in improving outcomes for what groups of service users, at what cost and why. At the moment, however, there are unrealised opportunities to maximise outcomes and to achieve cost savings, and commissioners, service managers and practitioners are making decisions in a situation of limited evidence. Advancing knowledge about reablement services may help prevent or delay people from requiring long-term care and support, reduce the risk of hospital admissions/re-admissions and maximise cost containment. This research is, therefore, both relevant and timely as it offers a unique opportunity to provide new evidence on different models of reablement services which will be of direct benefit to NHS managers, commissioners and their partner organisations: local authorities, third sector and independent providers.

## Additional file

Additional file 1: Questionnaire. (DOCX 128 kb)
