# Supplementary material for: Models of reablement evaluation (MoRE): a study protocol of a quasi-experimental mixed methods evaluation of reablement services in England
Source: BMC Health Serv Res. 2016 Aug 11;16:375. doi: 10.1186/s12913-016-1600-6 (PMC4981964; doi:10.1186/s12913-016-1600-6)
Supplement: Additional file 1: — Questionnaire. (DOCX 128 kb) [file 12913_2016_1600_MOESM1_ESM.docx]

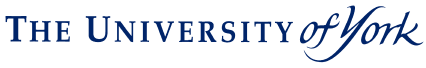

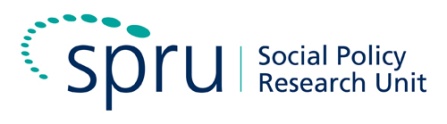


#

**Evaluating Models of
RE-ablement services**

**MoRE National Survey of Reablement Services**

HOW TO COMPLETE THE SURVEY^[[1]](#footnote-1)^

This survey is about the [NAME OF SERVICE] reablement service in the [LA/CCG NAME] that you manage. You will move through the survey automatically as you answer each question. Sometimes we will ask you a question that must be answered before you move on to the next section. This is so that we only ask you questions that are relevant to your service.

There are six parts to the survey. These are about:

1: Organisation and structure of your reablement service

2: Staffing

3: Referral and assessment

4: The type and reach of your reablement service

5: Assessing service users’ goals and progress

6: Funding and cost of the reablement service

The survey will take between 15 and 20 minutes. You do not need to complete it in one sitting; you will be able to close it and start again where you left off.  If you want to look again at your answers or change anything, use the 'back' button at the bottom of the page. After submitting your completed survey, you will be able to see the answers you gave and download a copy.

We hope you enjoy the survey.

Thank you so much for helping build this national picture of reablement services.

Closing date: [DATE]

Please be aware that if you do not press the submit button, we will still be able to see the information that you have provided after the closing date.

This questionnaire asks about the [NAME OF SERVICE]. If this is not correct, please type in the right name below.

- The correct name of my service is ____________________

**SECTION 1: ORGANISATION AND STRUCTURE OF YOUR REABLEMENT SERVICE**

**Q1.1 How would you describe the organisation you work in?** (select one option)

- Local authority social services department (1)
- Health service organisation (2)
- Joint health service and social services organisation (3)
- Private provider (for profit) (4)
- Voluntary provider (not for profit) (5)
- Social enterprise (6)
- Other (please provide details) (7) ____________________

**Q1.2 How would you describe [NAME OF SERVICE]?** (select one option)

- The service is wholly 'in-house' to this organisation (1)
- The service has both 'in-house' and contracted out elements (2)
- The service is wholly contracted out to another organisation (3)
- Other (please provide details) (4) ____________________

Answer If: How would you describe [NAME OF SERVICE] (select one)? The service has both 'in-house' and contracted out elements Is Selected

**Q1.3 Which elements of your service are contracted out?** (select all that apply)

- Assessing people for reablement eligibility (1)
- Assessing service users for a reablement plan (5)
- Health care input to service (2)
- Reablement input to service (3)
- Other (4) ____________________

Answer If: Which elements of your service are contracted out? (select all that apply) q://QID83/SelectedChoicesCount Is Greater Than or Equal to 1

**Q1.4 What type of contracts do you have with the contractors?** (select all that apply)

- Block (1)
- Spot (2)
- Outcome-based (3)
- Framework agreement (4)
- Other (please provide details) (5) ____________________

Answer If: How would you describe the reablement service you manage? The service is wholly contracted out to another organisation Is Selected

**Q1.5 What type of contract do you have with the contractor?** (select all that apply)

- Block (1)
- Spot (2)
- Outcome-based (3)
- Framework agreement (4)
- Other (please provide details) (5) ____________________

**Q1.6 How is [NAME OF SERVICE] operated and managed**? (select one option)

- As a separate reablement service (1)
- As part of an intermediate care service (2)
- As part of a home care service (3)
- As part of another service (please write in which service) (4) ____________________
- In another way (please provide details) (5) ____________________

**Q1.7 What are the objectives of your service?** (select all that apply)

- To help people regain everyday living skills (1)
- To reduce people's need for ongoing home-care (social/domiciliary care) (2)
- To prevent admission to long-term care when people are at risk of it (3)
- To prevent people staying in hospital for longer than is necessary (4)
- To prevent admission to hospital when people are acutely ill (5)
- Other (please give details) (6) ____________________

Answer If: What are the objectives of your service? (select all that apply) q://QID6/SelectedChoicesCount Is Greater Than 1

**Q1.8 What is the MAIN overall objective of your service?** (select one only)

- To help people regain everyday living skills (1)
- To reduce people's need for ongoing home-care (social/domiciliary care) (2)
- To prevent admission to long-term care when people are at risk of it (3)
- To prevent people staying in hospital for longer than is necessary (4)
- To prevent admission to hospital when people are acutely ill (5)
- Other (please give details) (6) ____________________

**Q1.9 Is [NAME OF SERVICE] organised into locality teams?**

- Yes (1)
- No (2)

Answer If: Do you have more than one reablement team in your service? Yes Is Selected

**Q1.10 How many locality teams do you have in [NAME OF SERVICE]?** (please write in number below)

**Q1.11 How long has [NAME OF SERVICE] existed in its present form?**

- Less than three months (1)
- Between three and twelve months (2)
- Between one and five years (3)
- More than five years (4)

**Q1.12 What age groups does the reablement service cater for?** (select one only)

- Adults over 18 years of age with no upper age limit (1)
- Adults between 18 and 65 years of age only (2)
- Adults over 65 years of age only (3)
- Other age range (please give details) (4) ____________________

**Q1.13 How would you describe** [NAME OF SERVICE]?

- It is a generic service that meets the needs of a wide range of people (1)
- It is a specialist service that meets the needs of a specific group of people (2)
- Other (please write in) (3) ____________________

Answer If: How would you describe the reablement service? (select one option only) A generic service that meets the needs of a wide range of people Is Selected

**Q1.14 Do you accept these client groups into your reablement service?** (select yes or no for each option)

|  | We support these client groups in our reablement service | |
| --- | --- | --- |
|  | Yes (1) | No (2) |
| People with dementia (1) |  |  |
| Younger disabled adults (up to the age of 65) (2) |  |  |
| People with learning disabilities (3) |  |  |
| People with brain injury (4) |  |  |
| People with sensory impairments (5) |  |  |

Answer If: Do you accept these client groups into your reablement service? (select yes or no for each option) - We support these client groups in our reablement service - Yes Is Greater Than or Equal to 1

**Q1.15 Do you have specialist pathways and/or protocols for this group?** (please select yes or no for each option)

|  | We support these client groups in our reablement service | |
| --- | --- | --- |
|  | Yes (1) | No (2) |
| People with dementia (1) |  |  |
| Younger disabled adults (up to the age of 65) (2) |  |  |
| People with learning disabilities (3) |  |  |
| People with brain injury (4) |  |  |
| People with sensory impairments (5) |  |  |

Answer If: How would you describe the reablement service? (select one option only) A specialist service that meets the needs of a specific group of people Is Selected

**Q1.16 Which special groups does [NAME OF SERVICE] serve?** (select all that apply)

- People with dementia (1)
- Younger disabled adults (up to the age of 65) (2)
- People with learning disabilities (3)
- People with brain injury (4)
- People with sensory impairments (5)
- Any other group (please provide details) (6) ____________________

**Q1.17 Do you provide reablement services to people if they have a carer (family member or friend) living with them?**

- Yes (1)
- No (2)

**Q1.18 Do you provide reablement services to people if they have a paid carer or personal assistant who lives with them?**

- Yes (1)
- No (2)

**Q1.19 Do you have any other explicit criteria for excluding people from [NAME OF SERVICE]?**

- Yes (please write in details) (1) ____________________
- No (2)

**Q1.20 Which days does [NAME OF SERVICE] provide a service to reablement clients?** (please select yes or no for each option)

|  | Yes (1) | No (2) |
| --- | --- | --- |
| Monday to Friday (3) |  |  |
| Saturday (4) |  |  |
| Sunday (6) |  |  |

Answer If: Which days does [NAME OF SERVICE] provide a service to reablement clients? (please select... - Yes Is Greater Than or Equal to 1

**Q1.21 Which hours do you provide a service to reablement clients?** (please select the option nearest to your service's hours)

|  | Yes (1) | No (2) |
| --- | --- | --- |
| Monday to Friday (3) |  |  |
| Saturday (4) |  |  |
| Sunday (6) |  |  |

**Q1.22 Does your reablement service cover the whole geographical area of [NAME OF LOCAL AUTHORITY/CCG]?**

- Yes (1)
- No (2)

**SECTION 2: THE TYPE AND REACH OF YOUR REABLEMENT SERVICE**

We are interested in what reablement services do with people to help them regain everyday living skills and continue to live at home. We are not asking here about things that your service does 'for' people but what you enable them to do for themselves.

**Q2.1 Please select the types of things your reablement service supports service users with, so that they can regain everyday living skills and remain at home (select all that apply)**

- Personal care (e.g. bathing/showering, dressing, toileting) (1)
- Domestic tasks (e.g. meal/drink preparation, doing laundry, ironing, cleaning) (2)
- Safety (e.g. awareness of safety in the home, preventing falls, providing aids and equipment, adaptations) (3)
- Information and signposting (e.g. pointing people towards financial advice, housing advice, other services/organisations that might help support them) (4)
- Getting around inside the home (e.g. using stairs, getting out of chairs) (5)
- Getting out and about outside the home (e.g. going shopping, travelling on the bus) (6)
- Re-engaging with social activities and friends (e.g. going to social clubs, taking part in usual hobbies) (7)
- Managing health-related needs (e.g. managing medication, managing stoma or catheter care) (8)
- Specific activities to help rebuild confidence and improve well-being (9)

Answer If: We are interested in what reablement services do with people to help them regain everyday... q://QID41/SelectedChoicesCount Is Greater Than 1

**Q2.2 Which of these makes up the majority of your work**? (please select one only)

- Personal care (e.g. bathing/showering, dressing, toileting) (1)
- Domestic tasks (e.g. meal/drink preparation, doing laundry, ironing, cleaning) (2)
- Safety (e.g. awareness of safety in the home, preventing falls, providing aids and equipment, adaptations) (3)
- Information and signposting (e.g. pointing people towards financial advice, housing advice, other services/organisations that might help support them) (4)
- Getting around inside the home (e.g. using stairs, getting out of chairs) (5)
- Getting out and about outside the home (e.g. going shopping, travelling on the bus) (6)
- Re-engaging with social activities and friends (e.g. going to social clubs, taking part in usual hobbies) (7)
- Managing health-related needs (e.g. managing medication, managing stoma or catheter care) (8)
- Specific activities to help rebuild confidence and improve well-being (9)

**Q2.3 Typically, for how many weeks do people receive input from your reablement service?** (Type in the number)

- Weeks (1) ____________________
- Don't know (2)

**Q2.4 Typically, how many total hours of reablement contact do people get while they are receiving your reablement service?** (Type in the number)

- Hours (1) ____________________
- Don't know (2)

**Q2.5 Can people receive your reablement service for more than six weeks in certain circumstances?**

- Yes (1)
- No (2)

If No Is Selected, Then Skip To End of Block

**Q2.6 In what circumstances do people receive the service for more than six weeks**? (select all that apply)

- Service user is not ready for discharge from reablement services but we expect improvement to continue (1)
- Service user requires on-going home care but care package has not been finalised (2)
- Service user requires on-going home care but onward service does not have capacity to accept onto their caseload (3)
- Other (please provide details) (4) ____________________

**Q2.7 How long can you extend the time limit of six weeks for?**

- Up to 7 days (1)
- Up to 14 days (2)
- Up to 21 days (3)
- As many days as needed depending on the service user's need (4)
- Other (please provide details) (5) ____________________

**Q2.8 Do you start to charge for the reablement service if people receive it for more than six weeks?**

- Yes (1)
- No (2)
- Don't know (3)

Answer If: If people do receive the reablement service for more than six weeks, do you start to charge for t... Yes Is Selected

**Q2.9 Which components do you charge for after six weeks?**

- Personal care only (e.g. washing, dressing) (1)
- Domestic tasks only (e.g. shopping, cooking, cleaning) (2)
- All care or services provided by the reablement team (3)
- Other (please give details) (4) ____________________

**SECTION 3: STAFFING**

This section is about the staff employed in your core reablement service. By core we mean the normal staff team working on a regular basis. This includes staff who are a regular part of the service, from any organisation. Please do not include people brought in from time-to-time to deal with high demand or for specialist input.

We are going to ask first about the number of people in your team and then about whole/full time equivalents.

**Q3.1 Please write in the total number of people in the core reablement team below.**

**Q3.2 How many whole time/full time equivalent posts are in the core reablement team?** (please write in the number below)

The next questions are about the type of staff in [NAME OF SERVICE]. We have put them into four groups. First we ask about social care practitioners, then therapists, then nursing staff and finally about 'other' staff.

**Q3.3 Which social care practitioners do you have working in the core reablement team in  [NAME OF SERVICE]?** (please select yes or no for each option)

|  | We have this type of practitioner in our core team | |
| --- | --- | --- |
|  | Yes (1) | No (2) |
| Reablement support worker (Bands 1/2/3) (1) |  |  |
| Social work assistant (2) |  |  |
| Senior social worker/team leader (3) |  |  |
| Care manager/case manager (4) |  |  |
| Home care worker (5) |  |  |
| Home care organiser (6) |  |  |
| Other social care staff (please write in below) (7) |  |  |

**Q3.4 Which therapists do you have working in your core reablement team in [NAME OF SERVICE]?** (please select yes or no for each option)

|  | We have this type of practitioner in our core team | |
| --- | --- | --- |
|  | Yes (1) | No (2) |
| OT assistant (Bands 2 to 4) (1) |  |  |
| Occupational therapist (Bands 5 to 7) (2) |  |  |
| Physiotherapy assistant (Bands 2 to 4) (4) |  |  |
| Physiotherapist (Bands 5 to 7) (5) |  |  |
| Other type of therapist (please write in below) (7) |  |  |

**Q3.5 Which health practitioners do you have working in your core reablement team in [NAME OF SERVICE]?** (please select yes or no for each option)

|  | We have this type of practitioner in our core team | |
| --- | --- | --- |
|  | Yes (1) | No (2) |
| Health care support worker (Bands 2 to 4) (1) |  |  |
| Registered nurse (Bands 5 to 7) (2) |  |  |
| Other type of health practitioner (please write in below) (4) |  |  |

**Q3.6 Are any other staff employed in your core reablement team in [NAME OF SERVICE]?**

- Yes (1)
- No (2)

Answer If: Please&nbsp;select any other staff working in your core reablement team. Speech and language therapist Is Selected

**Q3.7 Please select any other staff working in your core reablement team.**

- Speech and language therapist (1)
- Podiatrist (2)
- Dietician (3)
- Consultant geriatrician (4)
- Pharmacist (5)
- Psychologist (6)
- Psychiatrist (7)
- Community psychiatric nurse (8)
- Mental health support worker (9)
- Sensory impairment specialist (10)
- Other (please write) (11) ____________________

**Q3.8 Is the operational manager of [NAME OF SERVICE] included in the staff you have told us about above?**

- Yes (1)
- No (2)

Answer If: Is the manager or leader of the reablement service included in the staff listed above? No Is Selected

**Q3.9 What is the manager's profession (write in below)?**

**Q3.10 Are there any other staff you call into [NAME OF SERVICE]  to meet special needs or high demand, as and when needed?**

- Yes, for special needs (1)
- Yes, for high demand (2)
- No (3)

Answer If: Are there any other staff you call into [NAME OF SERVICE] to meet special needs or high d... Yes, for special needs Is Selected Or Are there any other staff you call into [NAME OF SERVICE] to meet special needs or high d... Yes, for high demand Is Selected

**Q3.11 Please select other staff you can call into the team to meet special needs or high demand when needed** (select all that apply)

- Reablement support worker (1)
- Occupational therapist (2)
- Social worker (3)
- Social care / home care assessor (4)
- Physiotherapist (5)
- Speech and language therapist (6)
- Podiatrist (7)
- Dietician (8)
- Consultant geriatrician (9)
- Pharmacist (10)
- Psychologist (11)
- Psychiatrist (12)
- Community psychiatric nurse (13)
- Mental health support worker (14)
- Visual impairment specialist (15)
- Other (please provide details) (16) ____________________

**Q3.12 When staff start to work in [NAME OF SERVICE], do they get training about ways of reablement working?**

- Yes (1)
- No (2)

Answer If: When staff start to work in [NAME OF SERVICE],&nbsp;do&nbsp;they get&nbsp;training about ways of reablement working? Yes Is Selected

**Q3.13 How is this training provided?** (select all that apply)

- On the job training (1)
- In-house training programme (2)
- External training programme (3)
- Other (please provide details) (4) ____________________

**Q3.14 Do staff working in [NAME OF SERVICE] get training updates about working in a reabling way?**

- Yes (1)
- No (2)

Answer If: Do staff working in [NAME OF SERVICE] get training updates about working in a reabling way? Yes Is Selected

**Q3.15 How is this training provided?** (select all that apply)

- On the job training (1)
- In-house training programme (2)
- External training programme (3)
- Other (please provide details) (4) ____________________

**Q3.16 Do you provide training for core team members to help them re-able people with special needs (for example, those with mental health issues or dementia)?**

- Yes (1)
- No (2)
- Not applicable, we don't provide services to people with special needs (3)

**SECTION 4: REFERRAL AND ASSESSMENT**

People seem to be referred to reablement services in two main ways:

Everyone referred for home care or domiciliary support is referred to the reablement service​

OR

Referral is selective - for example, people are referred when they are discharged from hospital, when they are felt to be at risk of admission to a care home, or when another professional feels they could benefit from reablement.

**Q4.1 Please choose the statement that is closest to what happens in your reablement services** (select one option)

- Everyone referred for home care (domiciliary) support is referred to our reablement service (1)
- People are referred to our reablement service in a selective way (2)
- Other (please provide details) (3) ____________________

**Q4.2 Who is able to refer people to your reablement service?** (select all that apply)

- Acute trust (hospital ward) (1)
- Early discharge team in an acute trust (2)
- Emergency department (3)
- Primary care (4)
- Primary care out-of-hours service (5)
- Social services emergency team (6)
- Social services intake team (7)
- Intermediate care bed-based unit (8)
- Intermediate care home-based services (9)
- People can refer themselves (10)
- Voluntary sector organisations (11)
- Other (please provide details) (12) ____________________

Answer If: How long has [NAME OF SERVICE] existed in its present form? Between one and five years Is Selected Or How long has [NAME OF SERVICE] existed in its present form? More than five years Is Selected

**Q4.3 How many referrals on average does your service receive each year?**

Answer If: How long has [NAME OF SERVICE] existed in its present form? Between three and twelve months Is Selected Or How long has [NAME OF SERVICE] existed in its present form? Less than three months Is Selected

**Q4.4 Typically, how many referrals does your services receive each month?**

**Q4.5 When a referral is made to your service, do you carry out your own initial screening before you admit them to the reablement service?**

- Yes (1)
- No (2)

**Q4.6 Once clients have been accepted into the [NAME OF SERVICE], what is the purpose of your first assessment?** (select all that apply)

- To carry out a full assessment of the person's reablement needs (2)
- To plan how the reablement service can meet the person's identified needs (3)
- To set specific goals for the person's reablement (4)
- To assess any other needs of the person referred (5)
- Other (please provide details) (6) ____________________

**SECTION 5: ASSESSING SERVICE USERS' GOALS AND PROGRESS**

There are different ways of assessing goals and progress in reablement. Some services set personalised goals for service users, some use standard measures for everyone to assess progress, and some use other methods.

**Q5.1 How would you describe the approach of [NAME OF SERVICE] to assessing goals and progress for service users?** (select all that apply)

- We set personalised goals for service users and use these to check progress (1)
- We use standard measures to assess everyone's progress (2)
- We use a different approach to assessing progress (please provide details) (3) ____________________

Answer If: How would you describe the approach of [NAME OF SERVICE] to assessing goals and progress for service users? (select all that apply) We set personalised goals for service users and use these to check progress Is Selected

**Q5.2 Are personalised goals set in partnership with the service user?**

- Yes, always (1)
- Yes, sometimes (2)
- No (3)

Answer If: How would you describe the approach of [NAME OF SERVICE] to assessing goals and progress for service users? (select all that apply) We set personalised goals for service users and use these to check progress Is Selected

**Q5.3 At what stage do you first set the personal goals?**

- Before reablement starts (1)
- Soon after reablement starts (2)
- Other (please provide details) (3) ____________________

Answer If: How would you describe the approach of [NAME OF SERVICE] to assessing goals and progress for service users? (select all that apply) We set personalised goals for service users and use these to check progress Is Selected

**Q5.4 Which staff usually set these goals?** (select all that apply)

- Reablement care workers (1)
- Social workers (2)
- Occupational therapists (3)
- Physiotherapists (4)
- Nurses (5)
- Other (6) ____________________

Answer If: How would you describe the approach of [NAME OF SERVICE] to assessing goals and progress for service users? (select all that apply) We use standard measures to assess everyone's progress Is Selected

**Q5.5 Which outcomes do you routinely assess for people who use your reablement service?** (select yes or no for all options)

|  | We assess | |
| --- | --- | --- |
|  | Yes (1) | No (2) |
| Mobility (1) |  |  |
| Quality of life (2) |  |  |
| General physical health (3) |  |  |
| General mental health (4) |  |  |
| Activities of daily living (5) |  |  |
| Social and personal outcomes (6) |  |  |
| Other (please give details) (7) |  |  |

Answer If: Which outcomes do you routinely assess for people who use your reablement service? (select yes or... - We assess - Yes Is Greater Than or Equal to 1

**Q5.6 How are these outcomes assessed?**

|  | We assess | |
| --- | --- | --- |
|  | Yes (1) | No (2) |
| Mobility (1) |  |  |
| Quality of life (2) |  |  |
| General physical health (3) |  |  |
| General mental health (4) |  |  |
| Activities of daily living (5) |  |  |
| Social and personal outcomes (6) |  |  |
| Other (please give details) (7) |  |  |

**Q5.7 When do you assess progress and outcomes?** (select all that apply)

- Before people receive their first reablement input to provide a baseline score/measure(1)
- On one or more occasions when people are receiving reablement (2)
- Towards the end of reablement (3)
- Some time after reablement has finished (please provide details) (4) ________________
- Other (please provide details) (5) ____________________

**Q5.8 What is the most common result for people at the end of input from your reablement service?**

- They are referred for FACS/Care Act eligibility assessment (1)
- They are discharged from the reablement service without any other care package (living independently) (2)
- They move into long-term care (3)
- They return to hospital (4)
- Other (please provide details) (5) ____________________
- Don't know (6)

**SECTION 6: FUNDING AND COST OF THE REABLEMENT SERVICE**

We need information about funding and cost so that we can calculate average costs for reablement services across the country. As with all the information in this survey, the details you provide in this section will not be shared with anyone outside the research team.

**Q6.1 Who funds your reablement service?**

- Local authority (1)
- Clinical commissioning group (CCG) (2)
- Joint local authority and CCG funding (3)

Answer If: How would you describe [NAME OF SERVICE]? (select one option) The service is wholly 'in-house' to my organisation Is Selected

**Q6.2 In the  financial year 2014-2015, what was the total spend for your reablement service?**

- Please give spend in £s (1) ____________________
- Don't know (2)

Answer If: How would you describe [NAME OF SERVICE]? (select one option) The service has both 'in-house' and contracted out elements Is Selected

**Q6.3 In the financial year 2014-2015, what was the total spend for your reablement service?**

- Total spend on in-house reablement service in £s (1) ____________________
- Total spend on contracts for contracted out elements £s (2) ____________________
- Don't know (3)

Answer If: How would you describe [NAME OF SERVICE]? (select one option) The service is wholly contracted out to another organisation Is Selected

**Q6.4 In the financial year 2014-2015, what was the value of the reablement contract?**

- Please give value of contract in £s (1) ____________________
- Don't know (2)

Answer If: How would you describe the organisation you work in? (select one option) Local authority social services department Is Selected Or How would you describe the organisation you work in? (select one option) Health service organisation Is Selected Or How would you describe the organisation you work in? (select one option) Joint health service and social services organisation Is Selected And In the&nbsp; financial year 2014-2015, what&nbsp;was the total spend for your reablement service? Don't know Is Selected Or In the financial year 2014-2015, what was the total spend&nbsp;for your reablement service? Click to write Choice 3 Is Selected

**Q6.5 What was the total budget for older people's services in [NAME OF LOCAL AUTHORITY/CCG] for the financial year 2014-2015?**

- Please give total budget in £s (1) ____________________
- Don't know (2)

Answer If: What is the total budget for older people's services in [NAME OF LOCAL AUTHORITY/CCG]? Please give total budget in £s Is Not Empty

**Q6.6 Approximately what percentage of that budget is spent on your reablement service?**

**Q6.7 What changes have there been to the budget for your reablement service in the last 12 months?**

- Budget has increased (1)
- Budget has decreased (2)
- Budget has stayed the same (3)

Answer If: What changes have there been to the budget for your reablement service in the last 12 months? (pl... Budget has increased Is Selected Or What changes have there been to the budget for your reablement service in the last 12 months? (pl... Budget has decreased Is Selected

**Q6.8 What are the reasons for these budget changes?**

- Changes in the configuration of the service (1)
- Changes in service user demand (2)
- Changes in the costs of service (3)
- Austerity measures/financial cuts (4)
- Other (please give details) (5) ____________________

We asked earlier about how many referrals your service got. The next question is about the total number of cases you provide reablement to.

Answer If: How long has your reablement service existed in its present form? Between one and five years Is Selected Or How long has your reablement service existed in its present form? More than five years Is Selected

**Q6.9 How many cases did [NAME OF SERVICE] provide reablement to in 2014 to 2015?** (please write in number below)

Answer If: How long has [NAME OF SERVICE] existed in its present form? Between three and twelve months Is Selected Or How long has [NAME OF SERVICE] existed in its present form? Less than three months Is Selected

**Q6.10 Typically, how many cases does [NAME OF SERVICE] provide reablement to in a month?** (please write in number below)

Answer If: How long has your reablement service existed in its present form? More than five years Is Selected Or How long has your reablement service existed in its present form? Between one and five years Is Selected

**Q6.11 In the last 12 months, has the ratio of staff to service users changed in [NAME OF SERVICE]?**

- We now have more staff per service user (1)
- We now have fewer staff per service user (2)
- Staffing has stayed about the same (3)

Answer If: In the last 12 months, has the number of staff per service user changed in your reablement service? We now have more staff per service user Is Selected Or In the last 12 months, has the number of staff per service user changed in your reablement service? We now have fewer staff per service user Is Selected

**Q6.12 What are the reasons for these changes?**

- Changes in demand for the reablement service (1)
- Changes in costs of services (2)
- Austerity measures/financial cuts (3)
- Other (please give details) (4) ____________________

If there is anything else you would like to tell us about your reablement service and the way it is organised and delivered, please use the space below.

Would you like to receive a summary of the national results of the survey later in the year?

- Yes (1)
- No (2)

1. *If you want to use this questionnaire in full, please contact the corresponding author to discuss the routing.* [↑](#footnote-ref-1)
